# Glass Fiber-Reinforced Polypropylene Composites with High Solar Reflectance for Thermal Insulation Applications

**DOI:** 10.3390/polym17030274

**Published:** 2025-01-22

**Authors:** Csenge Vámos, Tamás Bárány

**Affiliations:** 1 Department of Polymer Engineering, Faculty of Mechanical Engineering, Budapest University of Technology and Economics, Műegyetem rkp. 3., H-1111 Budapest, Hungary; csenge.vamos@furukawaelectric.com; 2 Furukawa Electric Institute of Technology Ltd., Késmárk u. 28/A, H-1158 Budapest, Hungary; 3 MTA-BME Lendület Lightweight Polymer Composites Research Group, Műegyetem rkp. 3., H-1111 Budapest, Hungary

**Keywords:** glass fiber-reinforced polypropylene composite, hierarchical porous layer, solar reflectance, reflective thermal insulation

## Abstract

Reflective thermal insulation layers can offer an energy-efficient strategy for preventing temperature rises by reflecting sunlight on surfaces. Our previous study presented a novel solvent-based method to prepare porous polypropylene (PP) with high solar reflectivity. However, the stiffness and strength of the neat porous PP were insufficient for thermal insulation applications, as mechanical loads from installation and environmental factors limit the applicability of such products. This paper addresses this gap by applying our solvent-based surface modification technology to glass fiber (GF)-reinforced PP composite sheets, creating a previously unexplored system. While the enhanced modulus and strength aligned with expectations, the micro- and nano-structured porous outer layers situated below the skin layer of the sheets, the refractive index mismatch between the PP matrix and the GF, and the size of the GF delivered a notable advancement in reflective thermal insulation performance. The combined effect of 30 wt% GF, nucleating agents, and surface modification resulted in a highly porous surface layer featuring spherulite sizes of 0.5–2.0 μm. With these combined effects, we achieved a modulus value of ~4 GPa, a tensile strength of 60 MPa, and an average solar reflectance of up to 94%. Thermal insulation performance measurements demonstrated that the registered inner temperature was lower by 24.1 °C compared to neat PP sheets. These combined effects demonstrate the potential of our solvent-based surface modification technology to develop cost-effective, porous PP composite sheets for efficient reflective thermal insulation.

## 1. Introduction

The EU’s new energy efficiency directive, effective since October 2023, tightens energy efficiency targets and accelerates the development of insulation solutions. By 2030, energy consumption must be reduced by 11.7% compared to 2020 forecasts [1]. High solar reflectivity plays a crucial role in mitigating intense solar radiation. Reflective thermal insulation techniques, valued for their cost-effectiveness and suitability for buildings, offer a practical solution by significantly reducing direct solar heat gain and lowering the energy demand of cooling. To further enhance this effect, researchers are developing materials with reflective and insulating properties across the solar spectrum, aiming to decrease overall energy consumption [2,3].

To enhance the reflectance of materials, traditional methods involve compounding organic polymers and reflective pigments, such as titanium dioxide (TiO_2_) [4], barium sulphate [5], silver [6], and silica [7] among others. However, forming aggregates of these fillers can limit achievable light reflectivity by overlapping the spatial intrinsic zones of pigments [4,5]. Also, poor compatibility between the polymers and reflective additives can lead to weak interfacial bonding, resulting in interfacial defects and decreased mechanical performance [6,7].

Introducing porous structures is another strategy for producing solar-reflective polymer materials, contributing to reducing indoor heat and, as such, the energy consumption in buildings exposed to solar radiation [8]. Such systems offer superior thermal insulation and solar reflective properties, as micropores were found to strongly scatter visible and near-IR radiation, while nanopores enhanced scattering in the UV and visible regions [9].

Numerous simulations were conducted to explore the impact of microscopic parameters of the porous material on optical properties [10,11,12]. Typically, in these simulations, scattering units containing circular voids with varying diameters (0.1 μm to 10 μm) were modeled using Mie scattering theory. According to Mie scattering theory, solar reflectivity is maximized by scattering units that induce a significant refractive index mismatch within the hierarchical porous structure [13]. These results revealed a significant size-dependent effect on scattering efficiency and wavelength. Air voids with a diameter of 1 μm demonstrated the highest weighted average scattering efficiency due to their optimal scattering band coverage and higher efficiency in the solar spectrum. Although larger air voids (2 to 10 μm) exhibited slight decreases in the weighted average scattering efficiency, they still provided substantial scattering across the entire solar range. Similarly, Mandal et al. [14] demonstrated that micropores (~5.0 μm) can efficiently scatter sunlight across the entire solar spectrum, while nanopores (~0.2 μm) enhance UV and visible scattering.

High porosity often comes along with poor mechanical strength and integrity [10,11,15]. Therefore, developing porous polymer structures with balanced porosity and mechanical properties is still a challenge and of growing interest worldwide.

In our previous studies [16,17], we introduced a new method utilizing phase separation to create a hierarchically structured (microscale- and nanoscale-patterned) porous layer on the surface of extruded polypropylene (PP) sheets. The treated sheets feature a dense top layer of PP, the so-called skin layer, over their porous surface layers. This skin layer also functions as a protective barrier, shielding the porous structure from environmental factors, such as dust and contaminants, as well as mechanical effects, including scratches and abrasions. The production process of these sheets involves several key stages, beginning with the incorporation of a nucleating agent of PP to promote the formation of an optimal pore structure. This is followed by sheet extrusion and solvent treatment of the extruded PP sheets for a specified duration, and a subsequent drying step to ensure complete solvent removal and optimal pore structure. The morphology of the created layers can be affected by the solvent-treatment parameters such as the immersion time, the temperature of solvent treatment, and the drying temperature [16]. The solvent-treated sheets exhibit a solar reflectance greater than 90% [18]. When the outer skin layer is removed, the porous PP layer shows a water contact angle (WCA) exceeding 160° [16]. Still, the basic mechanical properties were not optimal for outdoor applications.

Glass fiber-reinforced polypropylene (GFPP) composites have been widely used in mass production for decades. Their high stiffness, strength, and durability make them ideal structural engineering materials for challenging service environments, including variations in temperature, humidity, corrosive conditions, and sustained loading [19,20,21]. Also, in light of the growing need for a circular economy, there is a growing need for GFPP composites that offer improved recyclability, enabling their reuse both as manufacturing waste and as end-of-life components [22,23].

This paper presents the production and the performance of solar light-reflecting glass fiber-reinforced PP (GFPP) sheets having a hierarchically structured porous layer and an outermost skin layer covering the surface of the porous layer. The application of GFPP is expected to improve the mechanical properties of solvent-treated sheets. The morphology of the porous layer (the size and size distribution of the spherulites, the thickness of the porous layer) and the orientation of the glass fiber (GF) were examined by scanning electron microscopy (SEM). The crystallization behavior of the samples before and after the solvent treatment was investigated by differential scanning calorimetry (DSC). The mechanical properties of the samples were investigated by tensile and impact testing. Total reflection spectra were obtained from spectrophotometry measurements. Indoor and outdoor experiments were conducted with a laboratory-based apparatus to demonstrate the reflective thermal insulation performance of porous PP composites.

## 2. Experimental Methods

### 2.1. Materials

The experiments were carried out with a PP extrusion grade homopolymer, Tipplen H681F, with a melt flow rate (MFR, 230 °C, 2.16 kg) of 1.7 g/10 min, produced and supplied by MOL Petrochemicals Co. Ltd., Tiszaújváros, Hungary. The ADK STAB NA-21E nucleating agent (NA), sourced from Adeka Corporation, Tokyo, Japan, was incorporated into the polymer at a concentration of 500 ppm. The FGCS 3540 short GF (Schwarzwälder Textil-Werke Heinrich Kautzmann GmbH, Schenkenzell, Germany), having an average length of 3 mm and an average diameter of 10 μm, was used. The amount of GF in the composites was 20, 30, and 40 wt%. The Exxelor PO 1020 (ExxonMobil Chemical, Hanover, Germany) maleated polypropylene (MAPP) coupling agent (density 0.9 g/cm^3^, with a melt flow rate of 112 g/10 min (190 °C/1.2 kg)) was used at 10 wt% relative to the amount of GF. Extruded PP sheets containing either GF or MAPP individually were also prepared as reference samples. In these sheets, the ratio of PP to GF or PP to MAPP was equivalent to the ratio in the PP composites that contained both GF and MAPP. The composition of samples and their designations are listed in Table 1.

### 2.2. Sample Preparation

For the production of the masterbatches of NA PP with 1 wt%, the additive powders and PP pellets were dry-mixed in advance and extruded with a corotating twin-screw extruder (Brabender Plasti-Corder, Duisburg, Germany, features: L/D ratio: 19, screw diameter: 20 mm), with a rod die. The temperature zones were set to 190, 200, 200, 200, and 200 °C from the hopper to the die. The temperature of the die was 200 °C, and screw speed was 20 min^−1^. The extruded masterbatch filaments were pelletized with an S330 granulator (Rapid, Bredaryd, Sweden).

1 mm thick sheets from the different compositions summarized in Table 1 were extruded with a corotating twin-screw extruder (Cernobbio, Italy, features: L/D ratio: 40, screw diameter: 32 mm) using a slot die. The width of the die was 250 mm, and the gap size was set to 1.2 mm. All components were fed in the hopper of the twin-screw extruder. Barrel zone temperatures were set to 200, 210, 210, 210, and 205 °C from the hopper to die, while the temperature of the die along the entire width was set to 205 °C. The throughput was 6 kg/h, and the screw speed was set to 60 rpm. A calender (QCAL-Quadro Calandra Linea R&D-OG; Trocellen, Italy) was used for drawing. The temperature of the calender was set to 30 °C.

The surface modification process was based on a solvent treatment. PP composite samples were cut from the extruded sheets and immersed into hot xylene (a mixture of isomers, purity 98%, VWR Chemicals, Dramstadt, Germany) for defined time periods. A detailed description of our technology for obtaining a hierarchically structured porous layer was discussed in our previous paper [15]. In this study, we immersed our samples at 125 °C for 60, 120, and 180 s and dried them at 30 °C for 24 h in an air-ventilated oven (UT6120, Thermo Fisher Scientific, Waltham, MA, USA), and then for 24 h in a vacuum oven (FCD-3000, Faithful, Shanghai, China) at 30 °C.

### 2.3. Characterization

Cross-section morphology of the sheets was characterized with SEM (JSM-IT 200, Jeol, Tokyo, Japan). Before SEM imaging, samples were cryo-fractured and sputtered with a thin gold layer for 50 s at 10 mA in a vacuum. The structural layer over the thickness of the extruded samples was examined with a Zeiss Axioscope polarized optical microscope (POM) equipped with a Leica DFC 320 digital camera (Wetzlar, Germany). The melting and crystallization behavior of the samples was analyzed using differential scanning calorimetry (DSC Q2000; TA Instruments, New Castle, DE, USA). The DSC samples were first heated from 30 °C to 220 °C at 10 °C/min. This temperature was maintained for 5 min to eliminate thermal and mechanical prehistory, then the samples were cooled to 30 °C at a rate of 10 °C/min. The degree of crystallinity (X_c_) was calculated with Equation (1):

(1) $$X_{c}=\left(\frac{\Delta H_{m}}{\Delta H_{0}}\right)\times 100$$

where $\Delta H_{m}$ and $\Delta H_{0}$ represent the measured melting enthalpy of the first heating cycle and the melting enthalpy of a 100% crystalline sample, respectively; $\Delta H_{0}$ was taken as 207 J/g [24].

The average fiber length after the extrusion was measured, based on the fibers isolated from the ash of the composite materials pyrolyzed in a furnace at 550 °C for about 120 min. Extracted fibers were dispersed on a glass slide by water and evaluated with an optical microscope (Olympus BX51, Olympus, Germany). The average fiber lengths and fiber length distributions were determined based on the length of 500 fibers.

The weight percent of the GF in the extruded composite samples was determined using the calcination technique following the EN ISO 1172 standard [25]. The specimen was placed in a furnace under an inert environment at 500 °C for 120 min, then dried in a desiccator. The GF weight percent was calculated with Equation (2):

(2) $$GF\;\%=\frac{m_{2}}{m_{1}}\times 100$$

where $m_{1}$ is the initial weight of the composite sample and $m_{2}$ is the weight of the remaining GF after calcination.

Tensile tests were performed on five specimens from each composition using a Z010 universal tensile testing machine (Zwick/Roell GmbH, Ulm, Germany) equipped with a 10 kN load cell. The gauge length was 40 mm, and the crosshead speed was 5 mm/min. Young’s modulus was determined in the strain range of 0.2–0.6% by fitting a straight line to the stress–strain curve. The characteristic values of yield stress, tensile strength, and elongation at break were also determined. The tensile tests were performed in parallel to the machine direction. Impact tests were conducted on ten specimens from each composition using a falling weight impact tester (Fractovis 9350, Ceast, Italy) equipped with a 20 mm diameter hemispherical dart. The impact velocity was 4.4 m/s. The force–time diagrams were recorded during the tests, from which the perforation energy ($E_{perf})$ was calculated with Equation (3):

(3) $$E_{perf}=\frac{E_{total}}{v}$$

where $E_{total}$ is the total absorbed energy during the penetration and *v* is the thickness of the sample.

The total reflectance of solvent-treated PP composite samples was measured with a PerkinElmer 1050 spectrometer equipped with a 150 mm diameter Labsphere closed integrating sphere. The measurements were performed in a wavelength region of 200–2500 nm with a resolution of 10 nm. Three different positions of each sample were randomly selected for measuring. The total solar reflectance (TSR) in the solar spectral range was calculated with Equation (4):

(4) $$TSR=\frac{\int_{200}^{2500}R\left(\lambda \right)I_{solar}\left(\lambda \right)d\lambda }{\int_{200}^{2500}I_{solar}\left(\lambda \right)d\lambda }$$

where $R\left(\lambda \right)$ is the reflectance of the samples at the wavelength from 200 to 2500 nm, and $I_{solar}$ is the ASTM G173 [26] Global solar intensity spectrum. The TSR results were used to evaluate the reflected solar energy of the samples.

**Figure 1:**
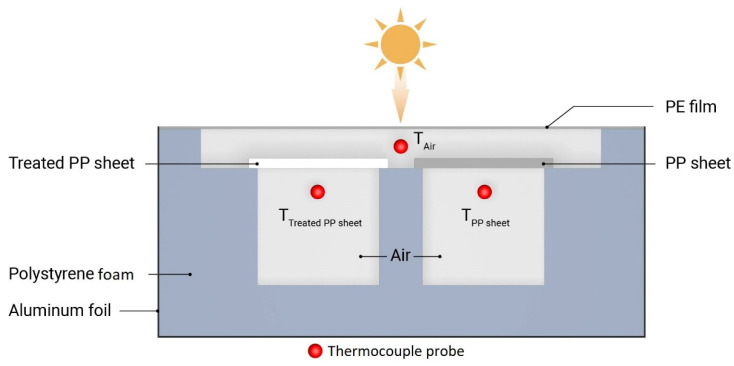
Schematic diagram of the apparatus for testing the reflective thermal insulation performance.

Indoor near-infrared light measurements were conducted to characterize the reflective thermal insulation ability of samples selected based on their TSR values, using a custom-designed apparatus (Figure 1). Two chambers were fabricated with a wooden frame, and the sides were covered with polystyrene foam sheets. In addition, the outer sides of the polystyrene foam sheet were sealed with aluminum foil to minimize heat convection and conduction. The top side of the chambers was covered with the selected samples. The inner temperature of the boxes was measured using K-type thermocouples. The boxes were irradiated from above with a 150 W light bulb, whose spectral distribution matched that of the solar spectrum well. The vertical distance between the lamp and the sample surface was 400 mm. A multi-channel temperature logger connected with two K-type thermocouples was used to detect the real-time temperatures inside the boxes. The test time was 1 h, and the internal temperature was recorded during the test.

The reflective thermal insulation performance of the samples was determined not only on an indoor test setup but also under the actual application conditions in natural solar light: tests were performed from 10:30 to 14:30 in Budapest on a sunny day on 18 July 2024.

A highly infrared-transparent low-density polyethylene (LDPE) foil was used to suppress the influence of the outdoor environment (for example, wind) on the test. Ambient temperature was measured with a thermocouple placed under the LDPE foil, which was also covered with an Al foil to prevent the tip of the thermocouple from being heated by direct sunlight.

## 3. Results

### 3.1. Cross-Sectional Morphology of Solvent-Treated PP Composites

**Figure 2:**
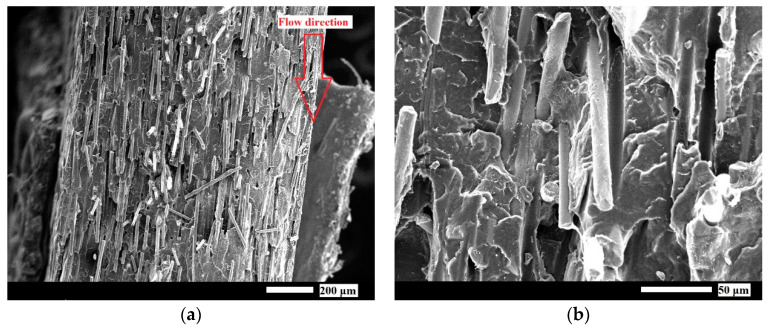

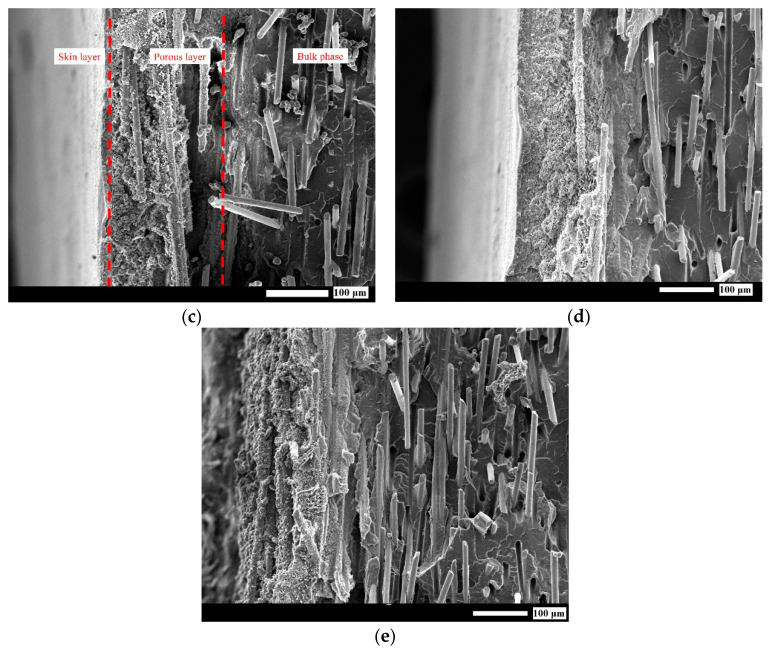
SEM images showing the cross-section of an extruded GFPP 30 sheet at (**a**) 100× magnification and at (**b**) 500× magnification. The (**c**) GFPP 20, (**d**) GFPP 30, and (**e**) GFPP 40 sheets after solvent treatment at 125 °C for 60 s at 200× magnification.

A cross-sectional morphology study was conducted using SEM to determine the impact of solvent treatment on the morphology of composites. Figure 2 shows both the cross-sectional SEM micrographs of the extruded GFPP 30 before and after the solvent treatment.

Figure 2a gives a representative image of the fiber orientation in the cross-section of the produced composite sheets. As expected, most fibers were oriented parallel to the flow direction, indicated with red arrows (Figure 2a,c). In Figure 2b, there were short pulled-out fibers on the fractured cross-sectional surfaces, leaving grooves at the fiber-matrix interface. However, it was also visible that most fibers exhibited good matrix adhesion, as shown by the retained PP matrix on the fiber surfaces. This good interfacial adhesion between the PP and GF can be attributed to MAPP—good interfacial adhesion is critical for the high tensile strength of the composites [27]. In the Supplementary Information, Figure S1 shows the weak interactions between the PP and GF without MAPP, resulting in longer pulled-out fibers and more pronounced grooves.

As was shown in our previous paper [16,18], and as can be seen in Figure 2c–e, solvent treatment led to a significant morphological change in the top layer of the extruded sheets. Under the skin layer, a hierarchically structured porous surface layer was composed of spherulites of different sizes formed due to simultaneous solvent evaporation and the recrystallization of PP. Supplementary information Table S1 presents the crystallinity changes in solvent-treated samples at different immersion times. Recrystallization led to a gradual increase in crystallinity, which consistently rose with longer immersion times. As the solvent treatment time increased, the ratio between the solvent penetration depth (solvent-affected layer) and the bulk phase thickness also grew, contributing to the overall increment in crystallinity.

In our previous study, we found that the solvent penetration depth into the bulk phase was a crucial parameter influencing the thickness and structure of the porous layer, the reduction of the bulk phase, and consequently, the optical and mechanical properties [15,17].

As such, we investigated the penetration depth of the solvent into the bulk extruded samples, as the remaining non-porous bulk layer, unaffected by the solvent, was expected to be responsible for the major load-bearing section. The solvent penetration depth was calculated by measuring the difference between the initial sheet thickness (1 mm) and the remaining bulk phase after solvent treatment. The thicknesses of both the bulk phase and the porous layer were determined through the analysis of cross-sectional SEM images.

**Figure 3:**
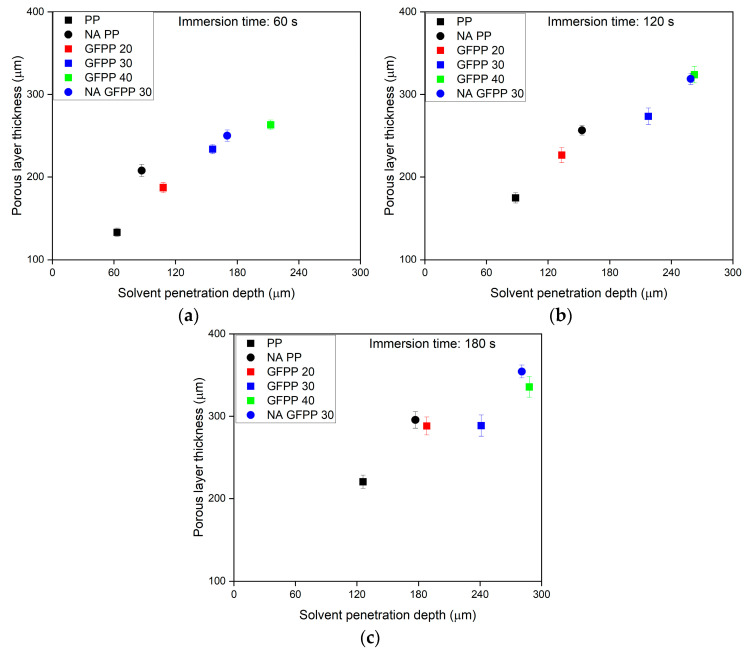
The thickness of the porous layers of the solvent-treated neat PP, GFPP, and NA GFPP 30 samples as a function of the solvent penetration depth. The samples were solvent-treated at 125 °C for (**a**) 60 s, (**b**) 120 s, and (**c**) 180 s.

Figure 3 presents the correlation between solvent penetration depth and porous layer thickness in samples treated for varying durations, with the results corresponding to one side of the samples.

The solvent penetration depth increased over the immersion time, similar to the previously studied PP samples [15]. The composition of the PP sheets significantly influenced the diffusion rate of the solvent into the extruded sheets. At a constant immersion time, the solvent diffusion rate into the bulk phase increased with a higher GF (and MAPP) content in the samples compared to the neat PP.

The individual effects of GF and MAPP content on solvent diffusion were examined on solvent-treated samples containing only GF (PP/GF 20, PP/GF 30, PP/GF 40) or only MAPP (PP/MAPP 2, PP/MAPP 3, PP/MAPP 4) (Supplementary information Figure S2). In PP/GF composites without MAPP, the solvent likely diffused more rapidly along the fiber surfaces into the bulk phase due to insufficient interfacial adhesion between the PP matrix and GF, compared to neat PP. In PP/MAPP samples, MAPP may act as a nucleating agent in the PP phase and facilitate faster solvent diffusion [28]. Analyzing the solvent penetration depth in the PP/GF, PP/MAPP, and GFPP composite systems separately demonstrated both GF and MAPP were found to enhance the rate of solvent diffusion.

Figure 3 also shows that the presence of the NA accelerated the solvent diffusion. The distinct super-morphological structures in the cross-section of the NA PP samples may facilitate faster solvent diffusion than neat PP. The POM images (Supplementary information Figure S3) indicated that the extruded PP sheets exhibited the expected core–shell morphology in the cross-section. The core layer is comprised of spherulites, while the outer shell layers consist of a highly oriented structure. In contrast, the POM images of the NA PP samples showed a micro-spherulitic structure throughout the cross-section without the core-shell morphology. It can be speculated that the thinner oriented-shell layer formed in the nucleated sheets led to deeper solvent penetration at the same immersion times. The combination of the NA-induced micro-spherulitic structure with GF and MAPP in the NA GFPP 30 samples resulted in the fastest solvent penetration, which can be beneficial as it enabled increased productivity during manufacturing of the sheets.

The thickness of the porous layer increased with a longer immersion time for a given composition, with a general correlation between the thicker porous layers and greater solvent penetration depth, irrespective of the sample composition. The size and size distribution of the spherulites within the porous layer were influenced by the composition of the PP composites, as illustrated in Supplementary information Figure S4. In solvent-treated neat PP, the size distribution of the spherulite was wide, with sizes ranging from approximately 23 μm to 0.5 μm. However, in the solvent-treated GFPP composite samples, the distribution narrowed and the spherulite sizes ranged between 10 μm and 0.5 μm. With increasing GF content, the spherulite size shifted towards smaller sizes. One may speculate that this was because during the extrusion process, the orientation of the GF can promote shear-induced nucleation and the formation of transcrystalline layers from the fiber surfaces [29]. These transcrystalline fronts may have disintegrated to some extent during solvent treatment. Still, the remaining order parts could act as self-nuclei during crystallization, resulting in the formation of smaller spherulites with a narrower size distribution.

In the solvent-treated NA PP and NA GFPP 30 samples, the effectiveness of the nucleating agent was clearly demonstrated by the fact that the size of the spherulites reduced drastically to 0.5–2.0 μm and was not affected by the GF content or immersion time. In these samples, the added heterogeneous nucleating agent governed the crystalline structure, promoting the formation of more crystal nuclei and thus smaller spherulites.

### 3.2. Solar Reflectance

The TSR results of the samples treated for different immersion times are summarized in Table 2 while Figure S5 in the Supplementary information presents their corresponding reflectance spectra.

Table 2 highlights the beneficial impact of solvent treatment and the introduction of GF and NA on increasing sunlight reflectivity. In non-treated samples, the addition of GF alone enhanced TSR, achieving approximately 33% for GFPP 40 compared to the ~12% TSR of neat PP. Presumably, both the refractive index mismatch between PP and GF and the small diameter (10 μm compared to the generally used 20 μm) of the GF resulted in increased sunlight scattering. While the introduction of NA alone did not significantly improve TSR, its combination with GF resulted in increased reflectivity, as demonstrated by the comparison between the GFPP 30 and NA GFPP 30 samples. In solvent-treated neat PP samples, TSR values ranged between 80% and 86%. Increasing the GF content to 30% raised TSR to approximately 90–92%, regardless of the immersion time. However, further increasing the GF content to 40% did not yield additional improvements. Beyond GF content, immersion time positively influenced the TSR of the GFPP samples. In contrast, solvent-treated NA PP and NA GFPP 30 samples exhibited TSR values exceeding 90% and 93%, respectively, even at the shortest immersion times. Notably, the TSR of NA GFPP 30 remained unchanged with longer immersion durations. These findings further reinforced that when NA is introduced, it played a dominant role in governing the resulting structure. The enhanced TSR observed in solvent-treated samples could be attributed to the previously presented spherulite size reduction in the upper layer.

In typical porous polymer structures described in the literature, the scattering units are circular air voids of varying sizes, and the scattering can be described by Mie theory. In contrast to these structures, which either feature a porous surface or exhibit porosity throughout the material, the collective solar light scattering behavior of our porous PP samples is more complex. First, our porous structure is buried beneath a skin layer, unlike the exposed porous surfaces or fully porous materials described in the literature. As such, the scattering may be attributed to various factors, including the characteristics of the skin layer, such as its thickness and surface roughness, as well as the properties of the underlying porous layer.

In our previous study, we investigated the effect of the skin layer on optical properties and found that it only slightly affected the reflectivity [18]. In our case, at the polymer–air interface in the porous PP layer, the refractive index mismatch between PP spheres (nPP = 1.49) and the surrounding air (air = 1.0) resulted in intense sunlight scattering. Increasing the porosity of the specific surface area by reducing the spherulite size led to higher scattering ability. The hierarchical spherulite structure also amplified scattering across the ultraviolet, visible, and near-infrared wavelengths, leading to a robust scattering effect [30,31]. The introduction of the GF contributed to the spherulite size reduction in the porous layer and created additional fiber/air and fiber/polymer scattering interfaces within the highly porous layer, which enhanced the total sunlight reflection and scattering.

**Figure 4:**
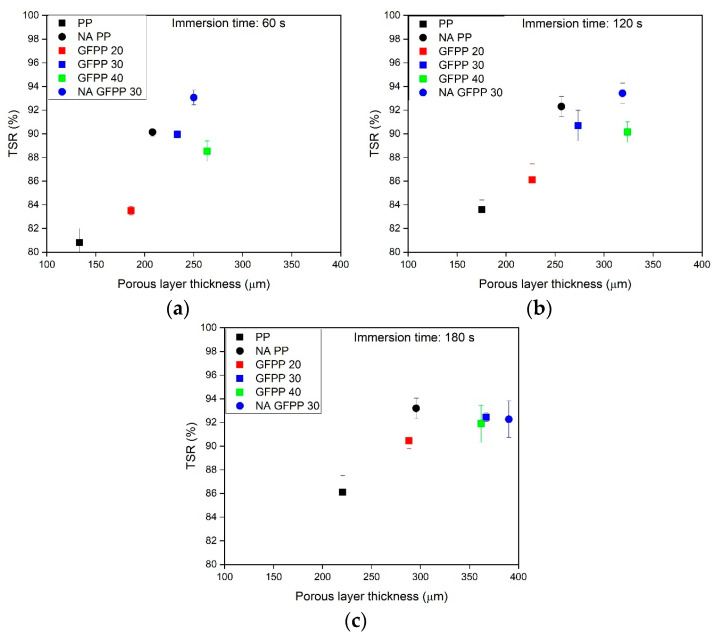
TSR of the solvent-treated samples as a function of porous layer thickness. The samples were solvent-treated at 125 °C for (**a**) 60 s, (**b**) 120 s and (**c**) 180 s.

In addition to the scattering from the interfaces, the spherulite itself could also have acted as a birefringent scattering center [31]. However, in our case, spherulites as scattering units could only have had a minor contribution as the light-scattering effect of the spherulites decreases proportionally with decreases in their size [31], which is the opposite of what we observed. Figure 4 illustrates the relationship between the TSR values and the thickness of the porous layers.

The TSR results of the solvent-treated samples increased significantly with increasing porous layer thickness, up to 250–300 μm. Beyond this point, further increases in thickness did not enhance TSR, which tended to plateau around 93%. This suggested that light cannot be efficiently reflected back from deeper than 250–300 μm, indicating that creating a thicker porous layer would not further improve optical properties.

The highest TSR values (93%) were observed in the NA GFPP 30 samples with an immersion time of 60 s (Figure 4a). With longer immersion times, no significant changes were observed (Figure 4b,c). The surface-treated NA GFPP 30 samples also showed that a porous layer thicker than 250 μm did not increase reflectivity.

### 3.3. Reflective Thermal Insulation Ability

**Figure 5:**
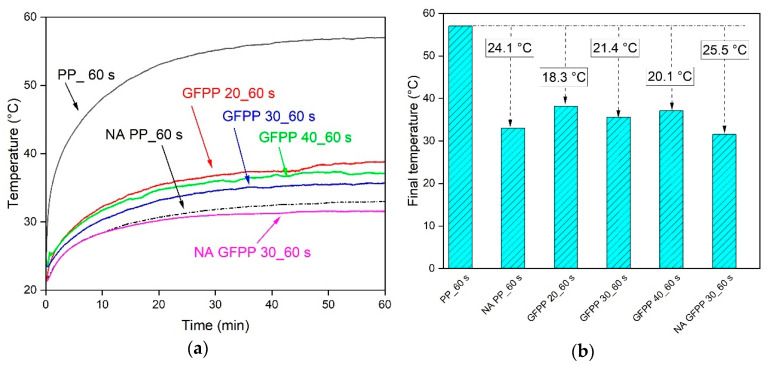
(**a**) Temperature tracking during the laboratory-based sun test and (**b**) final temperatures inside the boxes.

Considering the TSR results, the reflective thermal insulation properties of the solvent-treated samples with the shortest immersion time (60 s) as the most promising candidates will be discussed in the following section. First, the indoor test of the samples was evaluated using a laboratory-based sun test. We recorded the changes in inner temperature for the two boxes covered with an extruded PP sheet and a solvent-treated GFPP composite sample. The registered inner temperatures of the boxes are shown in Figure 5a, and the final inner temperature after 1 h is shown in Figure 5b.

The final temperature in the device covered with an extruded neat PP sheet was the highest, around 57 °C. The final temperatures of the solvent-treated GFPP composite samples, treated for 60 s, showed excellent alignment with the previously shown TSR results.

The registered inner temperature was lower by 24.1 °C for NA PP_60 s, 18.3 °C for GFPP 20_60 s, 21.4 °C for GFPP 30_60 s, 20.1 °C for GFPP 40_60 s, and 25.5 °C for NA GFPP 30_60 s.

**Figure 6:**
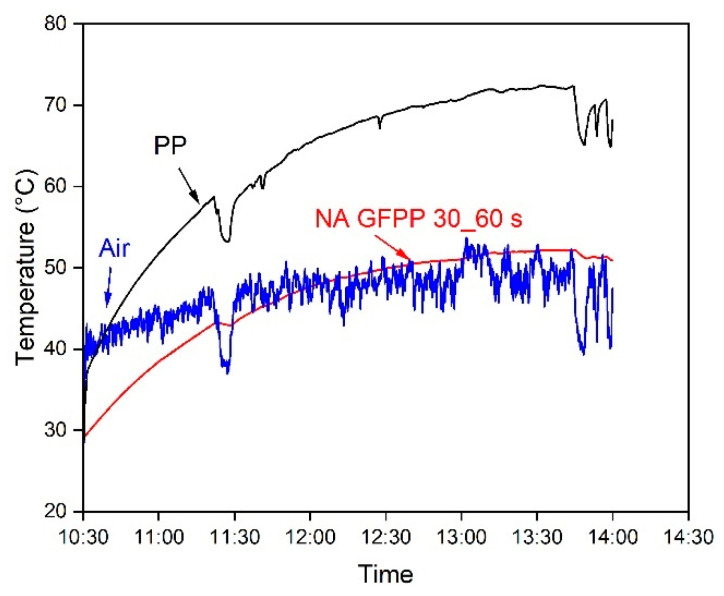
The recorded temperature of NA GFPP 30_60 s, ambient air, and neat PP.

To measure the reflective thermal insulation effect of the solvent-treated NA GFPP 30_60 s composite under more realistic conditions, we put the testing setup on the roof of a building in Budapest, where it was directly exposed to sunlight. The temperature data were recorded from 10:30 to 14:00 on 18 July 2024. The results are shown in Figure 6.

The average inside temperature of the chambers covered with NA GFPP 30_60 s was 46 °C, compared with the ambient air (~48 °C) on average between 11:00 and 14:00, after exposure to direct sunlight. In contrast, the temperature of the box covered with neat PP extruded sheet increased significantly, reaching above 67 °C under peak solar irradiation. The selected sample prevented the temperature rise far more than the reference extruded PP sheet under sunlight. The sample received significant solar irradiance, which was a clear sign of reflective thermal insulation ability.

### 3.4. Mechanical Properties

As the solvent treatment induced morphological changes and reduced the thickness of the bulk cross-section, it was crucial to evaluate its effect on the mechanical properties.

The actual glass fiber content and the average fiber length of the GFPP sheet samples are presented in Table 3. As expected, the average fiber length decreased with increasing glass fiber content.

**Figure 7:**
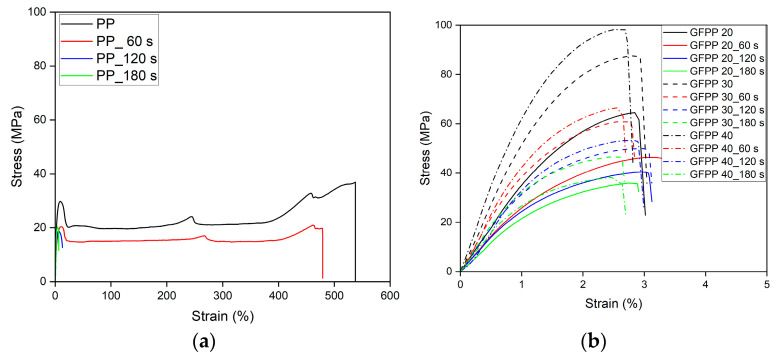
Typical stress–strain curves of solvent-treated (**a**) PP and (**b**) GFPP samples.

The impact of the solvent treatment on the mechanical properties of the GFPP composite samples was investigated with varying GF content and immersion times. Typical stress–strain curves of the samples treated for different immersion times are shown in Figure 7.

An increase in the immersion time during solvent treatment primarily caused significant changes in the failure mode of PP samples, as shown in Figure 7a. A ductile-to-brittle transition was observed with increasing immersion time, accompanied by a dramatic decrease in elongation at break. The stress–strain curves for the PP and GFPP samples differed significantly due to the presence of GF. GFPP composite samples exhibited brittle fracture behavior (Figure 7b), which is commonly attributed in the literature to stress concentrations at the fiber ends. These stress concentrations induce matrix cracking and, ultimately, matrix failure [32].

**Figure 8:**
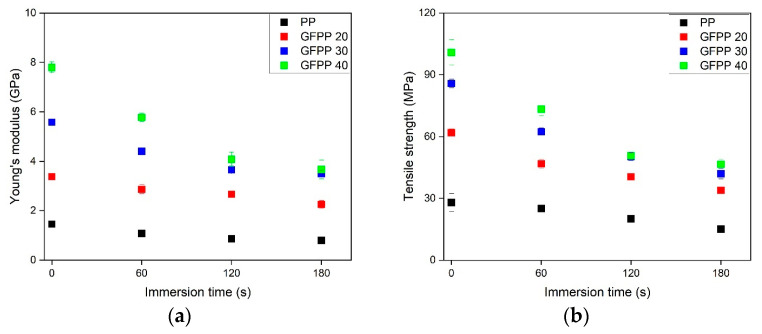
The effects of fiber content and immersion time on (**a**) the stiffness and (**b**) the tensile strength of the solvent-treated samples.

The results of the tensile tests are shown in Figure 8. The non-treated samples of 0 s immersion time represent the reference samples.

As shown in Figure 8a,b, adding GF met the expectations and significantly increases the stiffness (Young’s modulus) and tensile strength. Specifically, Young’s modulus increased from 1.5 GPa to approximately 8.0 GPa, while tensile strength improved from ~30 MPa to 100 MPa within the tested composition range. These findings aligned well with the literature data. Pukánszky et al. [33] investigated injection-molded GF-reinforced polypropylene (GFPP) with varying GF content from 0 to 50 wt%. Their study reported that increasing GF content to 30 and 40 wt% resulted in Young’s modulus values of approximately 6 GPa and 8 GPa, respectively. Furthermore, they emphasized the effect of maleic anhydride-grafted polypropylene (MAPP) as a coupling agent on tensile strength. GFPP samples containing 30 wt% GF exhibited ~40 MPa tensile strength without MAPP, which increased to ~70 MPa when MAPP was used.

As expected, the solvent treatment with longer immersion times resulted in decreased Young’s modulus and tensile strength values. This reduction was attributed to the reduced thickness of the bulk phase, as the solvent diffused deeper in the treated samples (Figure 3), thereby reducing the effective load-bearing cross-section and affecting the mechanical properties negatively. Despite these decreasing trends, the GFPP 30 and GFPP 40 samples, even after the longest immersion time of 180 s, still exhibited significantly higher Young’s modulus and tensile strength values than the untreated neat PP.

The deformability of the composites is crucial for practical applications. However, it is well known that while reinforcing fibers increase stiffness, they reduce deformability and toughness. Table S2 in the Supplementary Information summarizes the elongation at break values of the solvent-treated composites as a function of immersion time. The highest deformation was observed in non-solvent-treated samples, although increasing GF content negatively impacted deformability. For the GFPP composites, both solvent-treated and non-treated samples exhibited low (~1%) elongation at break because of the matrix fracture.

**Figure 9:**
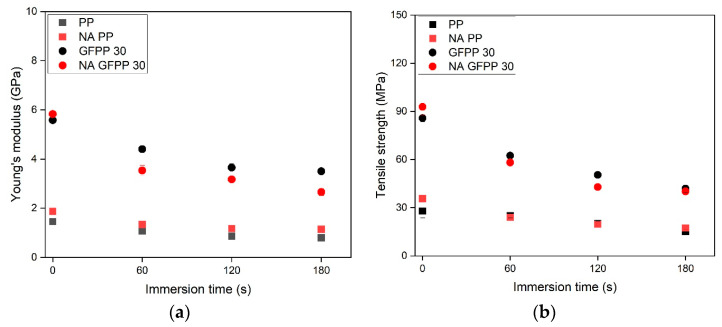
The effects of fiber content, NA, and immersion time on (**a**) the stiffness and (**b**) the tensile strength of solvent-treated samples.

The effect of applied heterogeneous NA on the mechanical properties of the solvent-treated NA PP and NA GFPP 30 samples is shown in Figure 9.

In Figure 8a, the non-treated NA PP resulted in a modulus increase of about 20% compared to the non-treated PP. A comparison of the non-treated GFPP 30 samples and the non-treated NA GFPP 30 samples showed that the nucleated PP composites exhibited a slightly higher Young’s modulus value (Figure 9a) and tensile strength (Figure 9b), as expected. Unexpectedly, the solvent treatment decreased the Young’s modulus and tensile strength values to a greater extent in the case of the NA GFPP 30 samples than in GFPP 30. This reduction was likely due to the combined effects of NA, GF, and MAPP, which accelerate solvent diffusion into the bulk phase, leading to a thinner load-bearing bulk layer. The elongation at break results (Supplementary Information Table S1) indicated that the presence of NA reduced sample deformability. Interestingly, while NA decreased elongation in samples without GF, it appeared to enhance elongation-at-break in GF-reinforced composites.

**Figure 10:**
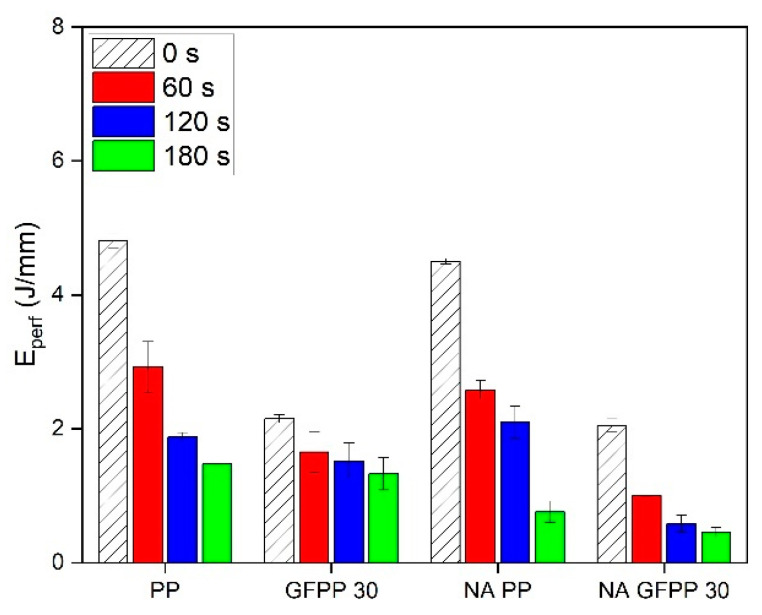
Perforation energy of the solvent-treated composite samples.

The composition and immersion time dependence of the perforation energy is presented in Figure 10.

For non-treated samples, the perforation energy showed a slight decrease in samples containing NA, while the addition of 30% GF resulted in a significant reduction in perforation energy. In general, the perforation energy decreased gradually with increasing immersion time. The perforation energy of NA GFPP 30 deteriorated the most with immersion time.

## 4. Conclusions

This study presents the characteristics of highly reflective extruded PP composite sheets for reflective thermal insulation applications, produced by using our novel solvent-based modification method. These sheets exhibited a distinctive structure, featuring a micro- and nano-structured porous layer situated beneath a skin layer—a configuration that, to the best of our knowledge, has not been previously reported by others in the literature.

The enhancement in reflectivity in the GFPP composites could be attributed to the addition of NA, GF, and MAPP, which reduced the spherulite size, narrowed their size distribution, and increased porosity, while also thickening the porous layer at a given solvent treatment time.

A combination of 30 wt% GF and nucleating agent resulted in a highly porous layer with spherulite sizes around 0.5–2.0 μm, achieving an average solar reflectance of up to 94%. In outdoor tests, the registered inner temperature of the NA GFPP 30_60 s sheets was 21 °C lower than that of the extruded neat PP sheets, demonstrating their suitability for reflective thermal insulation applications.

The solvent-treated GFPP composites exhibited a significant increase in stiffness and tensile strength compared to the surface-treated PP samples without GF. However, the solvent treatment reduced the thickness of the bulk phase, thereby decreasing the load-bearing cross-section with longer immersion times, which worsened the mechanical properties, including Young’s modulus and tensile strength. The modulus of the NA GFPP 30_60 s sample solvent-treated with the shortest immersion time was ~4 GPa, and its tensile strength was ~60 MPa.

We believe that applying our solvent-based surface modification technology to GFPP composite sheets will advance the development of large-scale, cost-effective, porous PP composites with enhanced mechanical properties, making reflective thermal insulation technologies more accessible.

## Figures and Tables

**Table 1:** Designations and compositions of extruded sheets.

Designation	GF Content (wt%)	MAPP Content (wt%)	NA Content (ppm)
PP	-	-	-
NA PP	-	-	500
PP/GF 20	20	-	-
PP/GF 30	30	-	-
PP/GF 40	40	-	-
PP/MAPP 2	-	2.5	-
PP/MAPP 3	-	4.3	-
PP/MAPP 4	-	6.7	-
GFPP 20	20	2.0	-
GFPP 30	30	3.0	-
GFPP 40	40	4.0	-
NA GFPP 30	30	3.0	500

**Table 2:** The TSR results of the solvent-treated samples.

	TSR (%)					
Immersion Time (s)	PP	NA PP	GFPP 20	GFPP 30	GFPP 40	NA GFPP 30
0 *	11.6 ± 0.6	12.4 ± 0.9	18.8 ± 1.4	22.7 ± 1.8	32.7 ± 2.2	28.7 ± 0.8
60	80.8 ± 1.2	90.1 ± 0.3	83.5 ± 1.6	89.9 ± 0.3	88.5 ± 0.8	93.0 ± 0.6
120	83.6 ± 0.8	92.3 ± 0.8	86.1 ± 1.3	90.7 ± 1.2	90.1 ± 0.8	93.4 ± 0.8
180	86.1 ± 1.4	93.2 ± 0.8	90.4 ± 0.6	92.4 ± 0.3	91.9 ± 1.5	92.3 ± 1.4

*: 0 s refers to the non-treated samples.

**Table 3:** Fiber characteristics before and after processing.

Designation	Nominal Fiber Diameter (μm)	Nominal Fiber Length (μm)		Actual Glass Fiber Content (wt %)
		Before Extrusion	After Extrusion	
GFPP 20	10	3000	1008 ± 278	21.1 ± 0.79
GFPP 30			800 ± 234	31.1 ± 1.10
GFPP 40			746 ± 199	40.7 ± 0.51
NA GFPP 30			812 ± 178	30.5 ± 0.31

## Data Availability

Data are provided within the manuscript or supplementary information files.

## Supplementary Materials

The following supporting information can be downloaded at: https://www.mdpi.com/article/10.3390/polym17030274/s1, Figure S1: SEM images showing the cross-section of an extruded PP/GF 30 sheet at (a) 100× magnification and (b) 500× magnification; Figure S2: The porous layer thickness of solvent-treated neat PP, PP/GF samples, and PP/MAPP samples as a function of solvent penetration depth. The samples were solvent-treated at 125 °C for (a) 60 s, (b) 120 s and (c) 180 s; Figure S3: POM images of (a) extruded PP sheet and (b) extruded NA PP sheet at 50× magnification; Figure S4: Spherulite size distribution along the porous layer of (a) PP, (c) GFPP 20, (e) GFPP 30, (g) GFPP 40, (i) NA PP, (k) NA GFPP 30 solvent treated for 60 s and (b) PP, (d) GFPP 20, (f) GFPP 30, (h) GFPP 40, (j) NA PP, (l) NA GFPP 30, solvent treated for 180 s; Figure S5: Reflectance spectra of (a) PP, (b) NA PP, (c) GFPP 20, (d) GFPP 30, (e) GFPP 40, (f) NA GFPP 30, treated with different immersion times; Table S1: Crystalline parameters of the extruded PP, GFPP, NA PP and NA GFPP samples; Table S2: The effect of fiber content and immersion time on the elongation at break of solvent-treated samples.
