# Supplementary material for: Glass Fiber-Reinforced Polypropylene Composites with High Solar Reflectance for Thermal Insulation Applications
Source: Polymers (Basel). 2025 Jan 22;17(3):274. doi: 10.3390/polym17030274 (PMC11820177; doi:10.3390/polym17030274)
Supplement: Supplementary file 1 [file polymers-17-00274-s001.zip › polymers-3432599-supplementary.pdf]

Table S1 Crystalline parameters of the extruded PP, GFPP, NA PP and NA GFPP samples

Designation	T_m (°C)	T_c (°C)	ΔH_m (J/g)	X_c (%)*
PP	167	113	80.52	38.9
PP_ 60 s	166	114	86.24	41.6
PP_ 120 s	164	114	89.83	43.4
PP_ 180 s	166	114	92.94	44.9
NA PP	165	129	85.28	41.2
NA PP_ 60 s	167	129	88.59	42.8
NA PP_ 120 s	167	129	90.87	43.9
NA PP_ 180 s	167	128	93.34	45.1
GFPP 30	166	119	47.50	38.2
GFPP 30_60 s	166	119	64.76	44.6
NA GFPP 30	166	130	61.29	42.2
NA GFPP 30_60 s	166	130	64.93	44.8

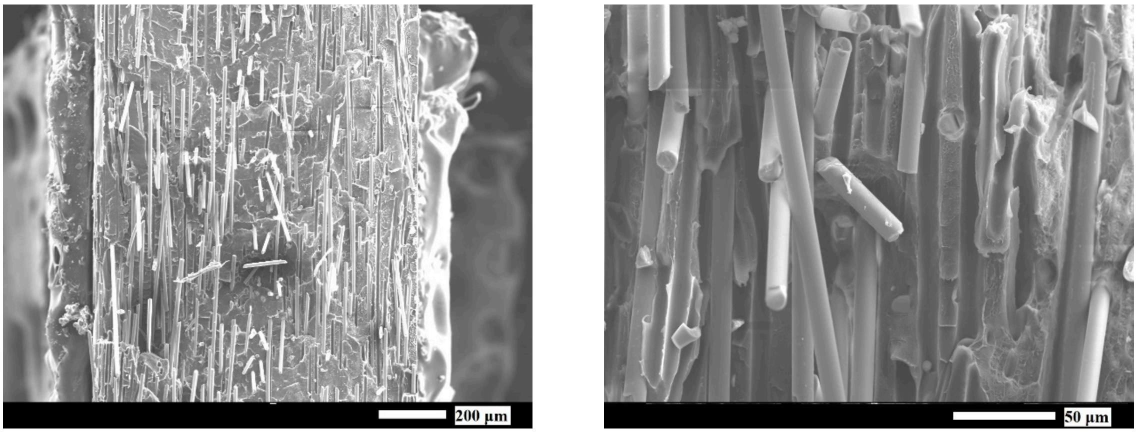

(a) (b)

Figure S1 SEM images showing the cross-section of an extruded PP/GF 30 sheet at (a) 100x magnification and (b) 500x magnification

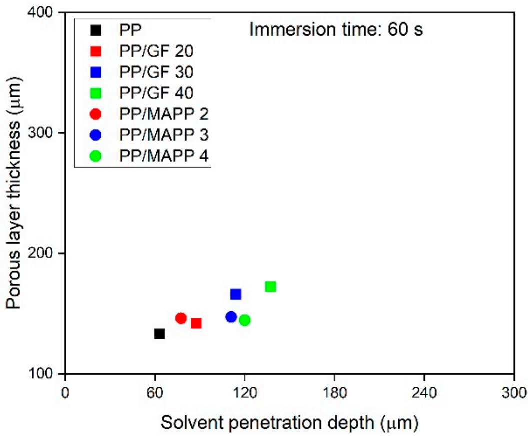

(a)

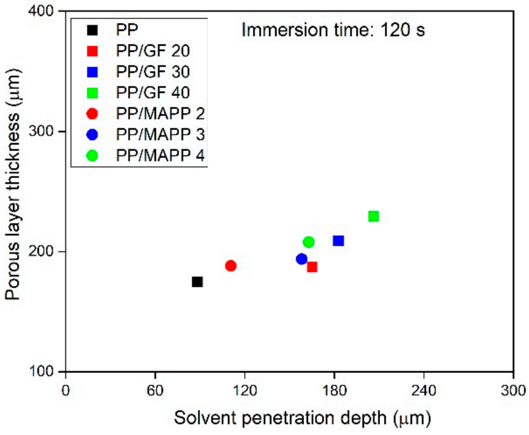

(b)

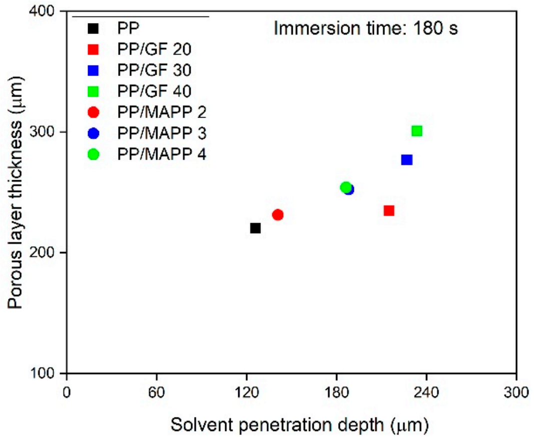

(c)

Figure S2 The porous layer thickness of solvent-treated neat PP, PP/GF samples, and PP/MAPP samples as a function of solvent penetration depth. The samples were solvent-treated at 125 °C for (a) 60 s, (b) 120 s and (c) 180 s

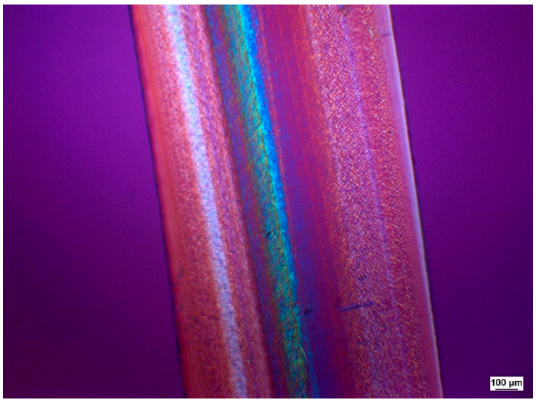

(a)

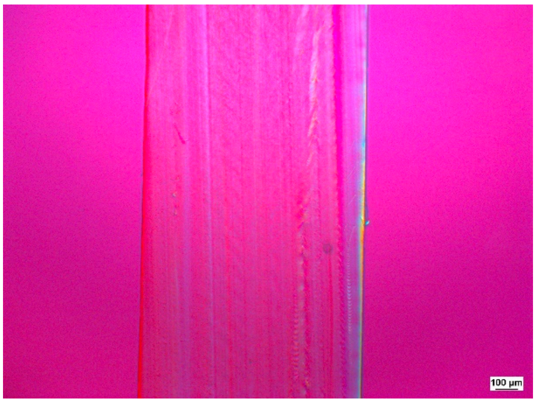

(b)

Figure S3 POM images of (a) extruded PP sheet and (b) extruded NA PP sheet at 50x magnification

Immersion time (s)

60 s | 180 s

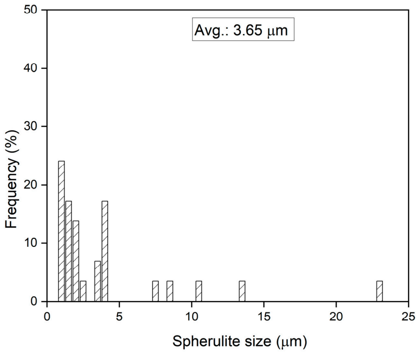

(a)

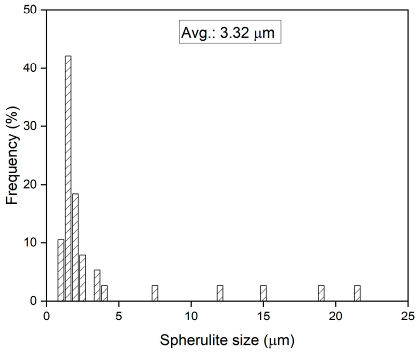

(b)

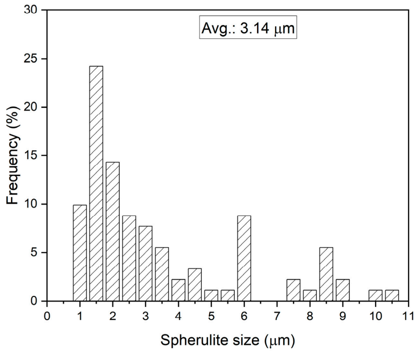

(c)

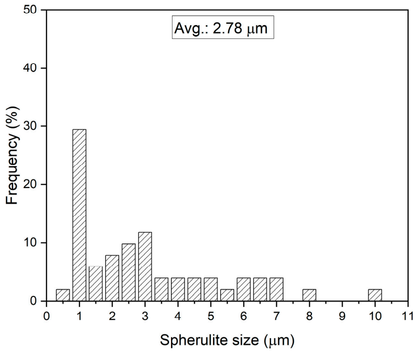

(d)

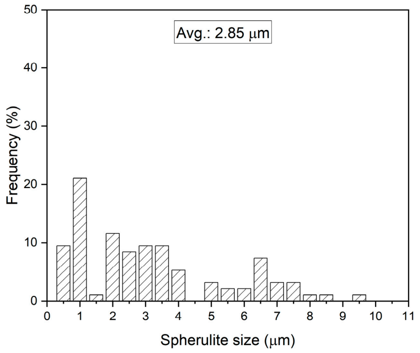

(e)

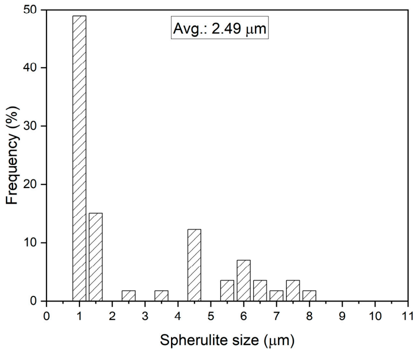

(f)

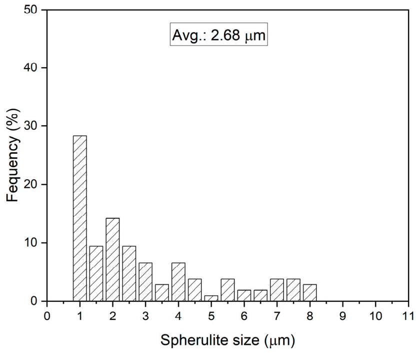

(g)

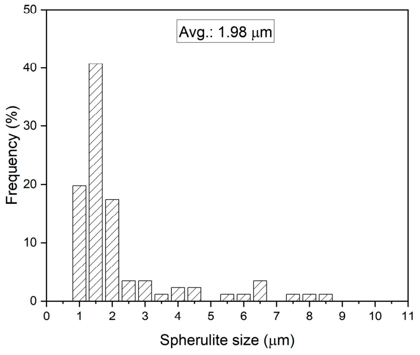

(h)

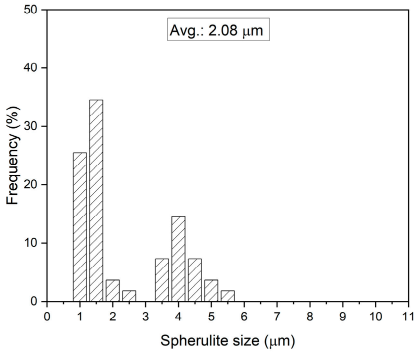

(i)

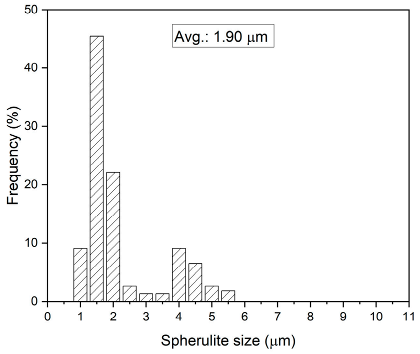

(j)

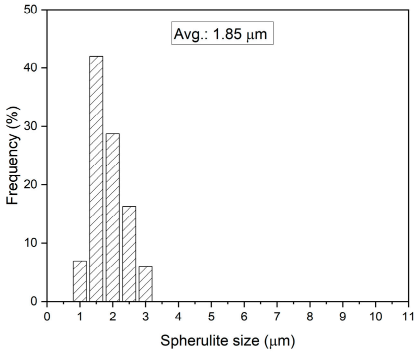

(k)

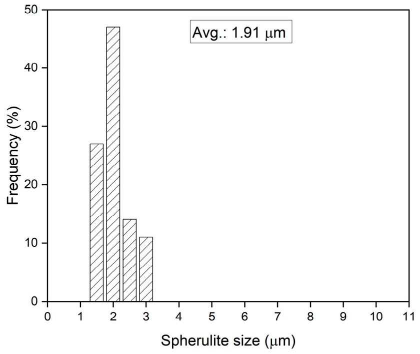

(l)

Figure S4 Spherulite size distribution along the porous layer of (a) PP, (c) GFPP 20, (e) GFPP 30, (g) GFPP 40, (i) NA PP, (k) NA GFPP 30 solvent treated for 60 s and (b) PP, (d) GFPP 20, (f) GFPP 30, (h) GFPP 40, (j) NA PP, (l) NA GFPP 30, solvent treated for 180 s

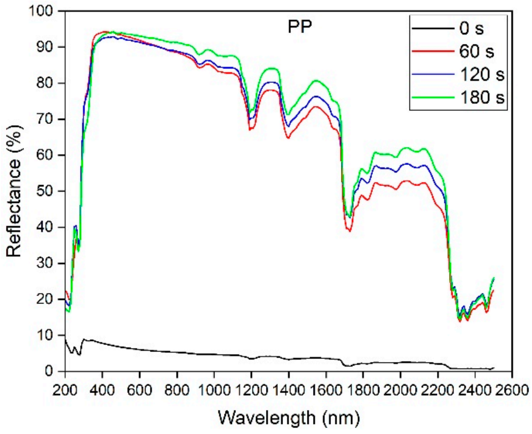

(a)

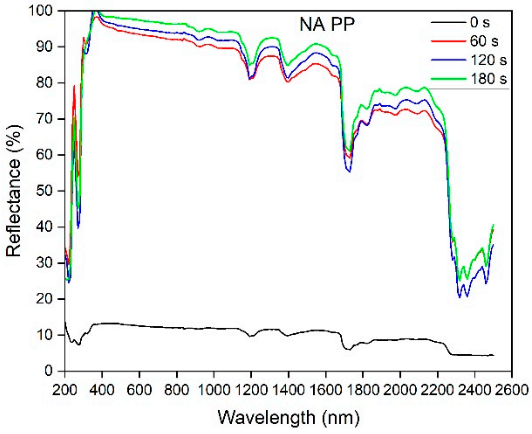

(b)

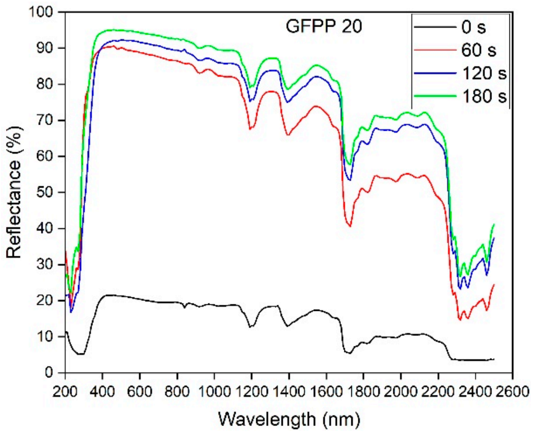

(c)

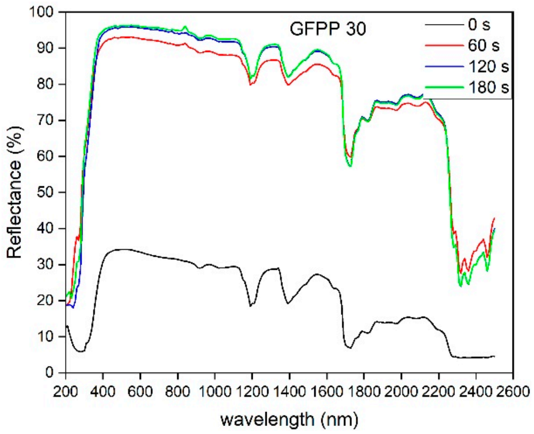

(d)

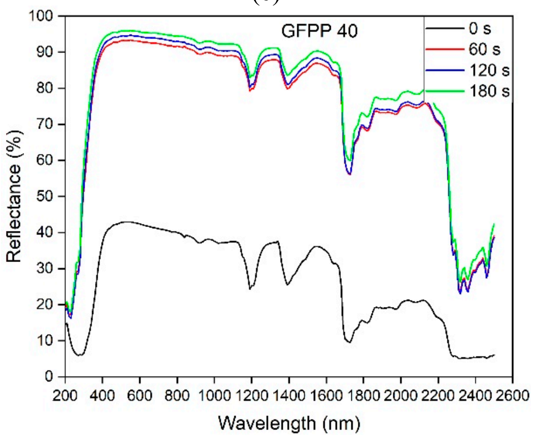

(e)

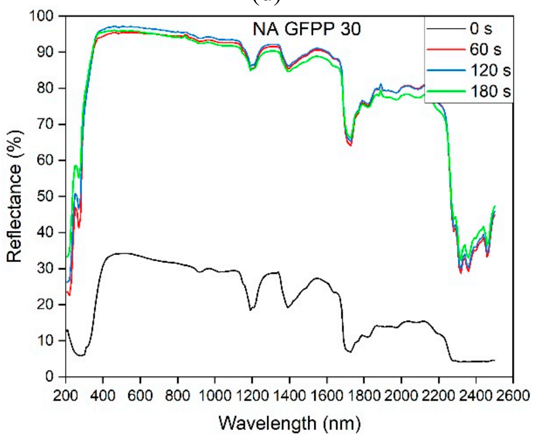

(f)

Figure S5 Reflectance spectra of (a) PP, (b) NA PP, (c) GFPP 20, (d) GFPP 30, (e) GFPP 40, (f) NA GFPP 30, treated with different immersion times.

Table S2 The effect of fiber content and immersion time on the elongation at break of solvent-treated samples

Immersion time (s)	Elongation at break (%)					
	PP	NA PP	GFPP 20	GFPP 30	GFPP 40	NA GFPP 30
0	464.6 ± 32.4	23.7 ± 5.1	1.0 ± 0.1	0.8 ± 0.1	0.8 ± 0.1	2.6 ± 0.1
60	425.8 ± 12.5	20.7 ± 1.9	1.0 ± 0.1	1.0 ± 0.2	1.0 ± 0.1	2.8 ± 0.1
120	287.3 ± 15.0	19.2 ± 0.6	1.1 ± 0.2	0.8 ± 0.1	0.9 ± 0.1	2.5 ± 0.1
180	25.6 ± 4.0	7.2 ± 0.5	1.1 ± 0.1	1.0 ± 0.1	0.9 ± 0.1	2.6 ± 0.1
